# Spatially heterogeneous thermal responses to urban form and greening: A multi-model analysis of seasonal and diurnal dynamics in beijing

**DOI:** 10.1016/j.isci.2026.115843

**Published:** 2026-04-24

**Authors:** Hui Zheng, Yudi Li, Chen Li, Xian Su, Junling Jin

**Affiliations:** 1 Guangxi Key Laboratory of Environmental Processes and Remediation in Ecologically Fragile Regions, Guangxi Normal University, Guilin 541006, China; 2 College of Environment and Resources, Guangxi Normal University, Guilin 541006, China

**Keywords:** Physical geography, Applied geography, Urban planning

## Abstract

Urban morphology and vegetation jointly regulate land surface temperature (LST) across seasonal and diurnal cycles, with implications for mitigating urban heat island (UHI) effects. Focusing on Beijing, we quantified the effects of two- and three-dimensional (2D/3D) urban form parameters and vegetation on LST using an integrated framework combining ordinary least squares (OLS), geographically weighted regression (GWR), extreme gradient boosting (XGBoost), and SHapley Additive exPlanations (SHAP). Results revealed pronounced nonlinear and spatially heterogeneous urban morphology-LST relationships and identified 250–300 m as the optimal explanatory scale. Compared with two-dimensional parameters, three-dimensional urban morphology exerted stronger control on LST, especially at night, whereas daytime cooling was dominated by the normalized difference vegetation index (NDVI). SHAP interaction analysis further showed that vegetation cooling and building-induced warming were jointly conditioned by urban form and ventilation across seasonal and diurnal contexts. These findings provide a structural basis for differentiated, scale-sensitive heat-mitigation strategies in high-density cities.

## Highlights

• Urban form-LST relationships are strongly scale-dependent and nonlinear
• Vegetation cools daytime LST, but its effect is constrained by urban form
• Three-dimensional urban morphology dominates nighttime heat retention
• Integrated morphology-greening-ventilation design is essential for heat mitigation

## Graphical abstract

**Figure:**
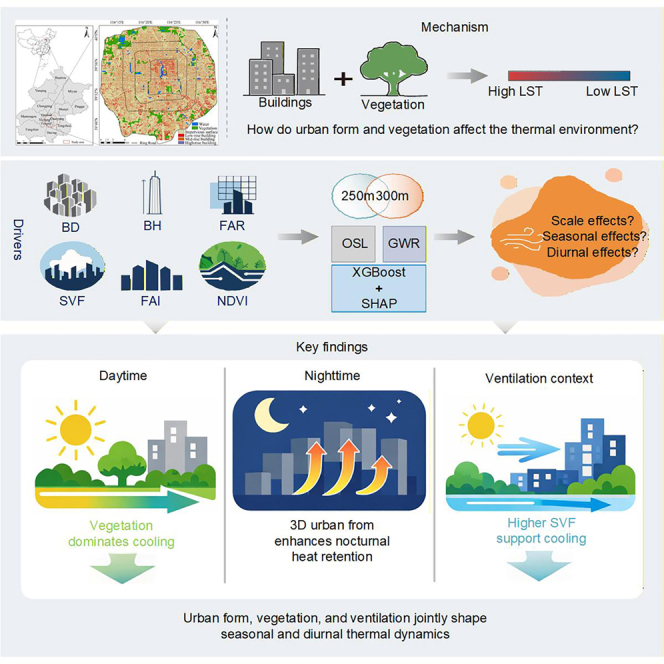

## Introduction

Against the backdrop of global warming and rapid urbanization, anthropogenic factors, such as urban population growth and urban expansion, have significantly affected the thermal environment of cities, thereby causing and exacerbating the urban heat island (UHI) effect,^1^^,^^2^ in which temperatures in urban areas are higher than those in the surrounding rural or suburban areas.^3^ Natural surfaces have a significant cooling effect owing to both transpiration and albedo variability^4^^,^^5^^,^^6^; conversely, artificial surfaces have a significant warming effect.^7^^,^^8^ Natural elements, such as water bodies, vegetation, and soil, are being gradually replaced by artificial elements, such as paved roads and windproof and heat-insulated buildings.^9^^,^^10^ This has resulted in larger and hotter UHIs, which can negatively affect the urban climate and public health,^11^ increase energy consumption and pollutant diffusion, reduce air quality and biodiversity, and hamper the sustainable development of cities.^12^^,^^13^ In recent years, the rapid increase of the urban population and shortage of available land have led to the continuous expansion of urban buildings—both horizontally and vertically.^14^^,^^15^ Consequently, urban buildings have undergone drastic changes, and optimizing the urban building patterns, reducing the negative effects of UHIs, and improving the urban thermal comfort have attracted considerable attention from both researchers and urban planners.^16^

In recent years, research on the relationship between urban morphology and land surface temperature (LST) has gradually shifted from single-indicator analyses to comprehensive comparisons of multidimensional urban morphological parameters (UMPs), leading to a more systematic understanding across different climatic contexts.^17^^,^^18^ From a climatic perspective, the thermal effects of UMPs exhibit significant regional heterogeneity.^19^ In high-density hot-humid cities in East and Southeast Asia, building density (BD) and floor area ratio (FAR) generally contribute to significant increases in LST,^20^^,^^21^ whereas building height and spatial enclosure exert more context-dependent influences by modulating ventilation and radiative exchange processes.^22^^,^^23^ In parts of mid-to high-latitude European cities, the sky view factor (SVF) and street-canyon geometry have been identified as key factors regulating diurnal variations in LST.^24^^,^^25^ Cross-climatic comparisons show that in arid or semi-arid cities, compact forms characterized by lower SVF and stronger enclosure enhance shading and reduce heat exposure, as observed in hot-arid cities of the United Arab Emirates.^26^^,^^27^ In temperate humid cities, however, the importance ranking of variables shifts; in Istanbul, FAR and the normalized difference vegetation index (NDVI) play a more dominant role, whereas SVF is relatively weaker.^28^^,^^29^ Moreover, multi-city evidence further indicates that both the direction and magnitude of thermal responses vary across urban types and city sizes.^30^^,^^31^ For example, the frontal area index (FAI) is negatively correlated with LST in some high-density Asian cities,but positively correlated under other climatic settings.^32^ Similarly, SVF shows positive correlations with LST in Athens and negative correlations in Berlin and Dalian.^33^ Global large-sample analyses also reveal significant scale effects between city size and the thermal environment, with differentiated expansion trajectories and climate-dependent vegetation cooling efficiency.^25^ However, existing global evidence remains fragmented across climate zones, urban densities, and methodological frameworks, and systematic cross-scale comparisons integrating 2D and 3D morphological parameters with vegetation factors under unified analytical settings are still limited.

Urban thermal environments are shaped by multiple interacting factors, including urbanization processes, climatic background, land-use change, and human activities. These macro-level drivers typically influence local thermal patterns indirectly by altering spatial form and surface cover structure.^34^^,^^35^^,^^36^ Existing studies on urban form have primarily focused on the two-dimensional (2D) and three-dimensional (3D) structural characteristics of quantifiable built environments, such as buildings.^30^ Vegetation, as an integral component of urban spatial form, is commonly represented by planar indicators such as the NDVI and can likewise be regarded as a 2D urban morphology parameter. Together with built structures, it constitutes the overall urban morphological system, although it regulates the thermal environment through energy and water exchange pathways distinct from those of built form.^37^^,^^38^^,^^39^ Therefore, it is necessary to examine the thermal regulatory role of vegetation under different temporal scenarios within a unified analytical framework of urban morphology. Vegetation is widely recognized as a key factor in mitigating the UHI effect.^40^^,^^41^ Its cooling capacity mainly relies on evapotranspiration and shading, leading to pronounced seasonal and diurnal non-stationarity.^23^ Previous studies have shown that the NDVI generally exhibits stronger cooling effects in summer and autumn due to enhanced evapotranspiration, whereas in winter its cooling effect may weaken—or even shift to a positive correlation with LST—because of reduced shading and changes in surface albedo.^38^^,^^42^ This implies that empirical relationships derived from a single season or temporal snapshot cannot be directly generalized to year-round strategies. Existing research has predominantly examined the independent effects of individual morphological parameters or vegetation indices, leaving their interaction mechanisms—particularly nonlinear and threshold-dependent behaviors across seasonal and diurnal contexts—insufficiently explored. As a result, systematic explanations of the synergistic effects between built form and vegetation remain limited.^43^^,^^44^

Owing to differences in geographical location, local climate, building form, and city layout, UMPs exert scale-based effects on UHIs,^18^^,^^32^^,^^45^ and the optimal urban morphological scale at which to study UHIs differs depending on the city. For Beijing, the optimal morphological scale at which to study UHI was 270 m^18^; for Wuhan, it was 180 m^32^; and for Xi’an, it was 200 m.^46^ Therefore, the urban morphological scale should be considered in regression models to examine the effects of drivers on LST.

Urban morphology and vegetation are closely related to thermal environmental changes,^20^^,^^47^ and their impacts on UHIs have been examined using various methods. For instance, climate models are widely used to study local urban climate mechanisms.^48^^,^^49^^,^^50^ The WRF-UCM, UrbClim, and MUKLIMO_3 models, which conduct large-scale simulations, have been used to study the impacts of urban morphology on the thermal environment.^51^^,^^52^ However, owing to the influence of the input data and model parameters, the uncertainty of their simulation results is relatively high. Geographic information systems (GIS) and remote sensing-based methods can be used to quantitatively analyze UHI effects,^18^ and ordinary least squares (OLS) and correlation analyses are widely used to study such effects.^51^^,^^53^ However, OLS is a global linear model that does not consider the impact of spatial heterogeneity, thereby generating biased results.^54^ To address this limitation, geographically weighted regression (GWR) has been introduced to capture the spatial non-stationarity of morphological effects.^55^ Nevertheless, numerous studies have shown that LST often responds to UMPs and vegetation factors in a markedly nonlinear manner, with potential threshold effects, indicating that linear or locally linear models alone remain insufficient. In recent years, machine learning approaches, including random forests, boosted regression trees, and extreme gradient boosting (XGBoost), have been employed to model such complex nonlinear relationships^33^; however, their “black-box” nature constrains mechanistic interpretation. The introduction of interpretable methods such as SHapley Additive exPlanations (SHAP) has provided new opportunities to quantitatively disentangle the relative contributions and interaction effects of different drivers.^56^

**Figure 1:**
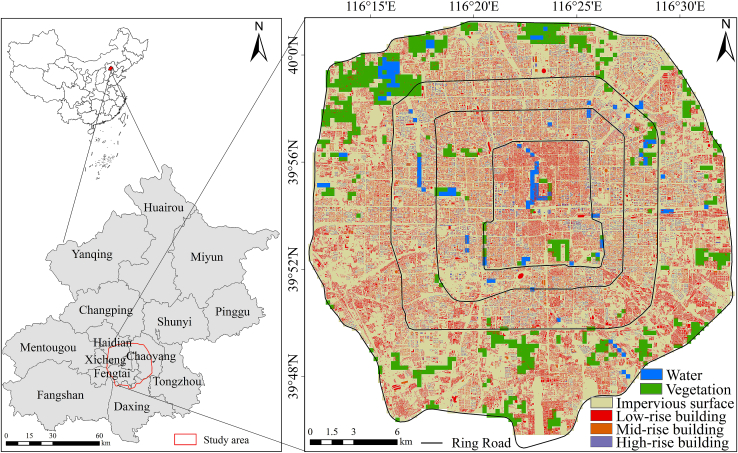
Location and scope of the study area and distributions of vegetation, water bodies, impervious surfaces, and building heights

**Figure 2:**
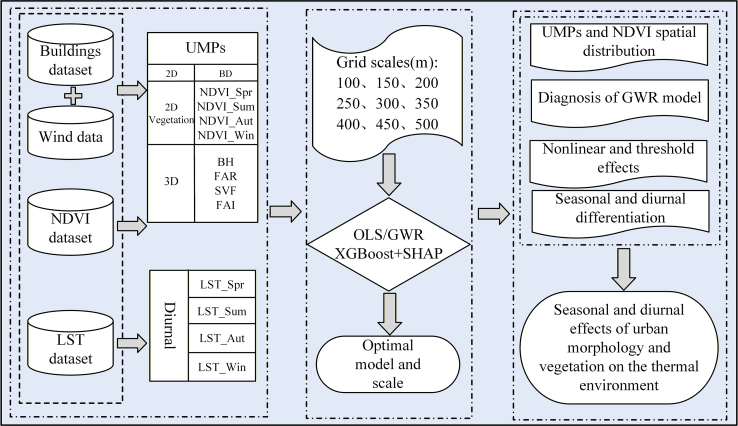
Research framework

To address these gaps, we examined the impacts of urban morphology on seasonal and diurnal differences in the thermal environment, using Beijing as a case study (Figure 1). To achieve this, the OLS, GWR, XGBoost, and SHAP models were constructed using data on building vectors, wind direction, LST, and the NDVI, following the research framework shown in Figure 2. The main contributions of this study are 3-fold: (1) Within a unified multi-scale framework, we integrate 2D and 3D UMPs with vegetation factors, and systematically identify their differentiated dominant mechanisms across seasonal and diurnal contexts, overcoming the limitations of single-dimension or single-temporal analyses. (2) By accounting for scale effects and spatial heterogeneity, we combine statistical regression with interpretable machine learning to quantify the nonlinear responses, threshold effects, and interactions of key drivers, thereby advancing the mechanistic understanding of urban thermal processes. (3) Using Beijing as a representative high-density, rapidly urbanizing city, we determine the optimal explanatory scales and spatiotemporal non-stationarity of key morphological variables, providing scale-sensitive empirical support for heat-risk-adaptive urban design and refined thermal management.

## Results

### Data and spatial distributions of LST

**Figure 3:**
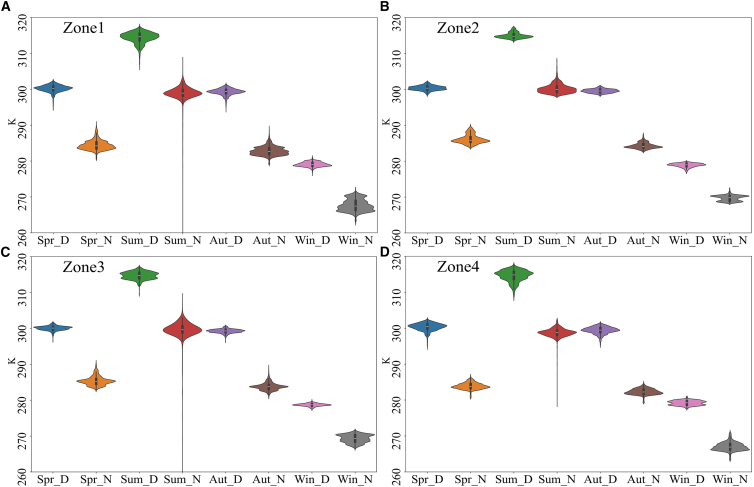
Distributions of diurnal land surface temperature (LST) in different zones in the study area (A), (B), (C), and (D) represent the results in Zone1, Zone2, Zone3, and Zone4, respectively. Spr_D, Spr_N, Sum_D, Sum_N, Aut_D, Aut_N, Win_D, and Win_N represent spring daytime, spring nighttime, summer daytime, summer nighttime, autumn daytime, autumn nighttime, winter daytime, and winter nighttime, respectively. K stands for the thermodynamic unit of LST, Kelvin. Data are shown as violin plots with embedded boxplots; the center line indicates the median, and the box indicates the interquartile range.

In each zone, the seasonal mean LST differed markedly between daytime and nighttime (Figure 3; Table S2). Zone 1 and Zone 3 exhibited similar LST distributions, reflecting their comparable urban functions and morphological characteristics within the inner urban area. Many LST outliers were present in the nighttime summer data, mainly for locations such as the Old Summer Palace, Chaoyang Park, and Metro Park, potentially because these locations contain large areas of vegetation and water. During nighttime, reduced transpiration limits evaporative cooling, whereas water bodies, owing to their high specific heat capacity, release stored heat more slowly.^57^ These contrasting thermal behaviors may contribute to the observed outliers. Differences in LST among the zones may be related to building patterns, vegetation coverage, and water distribution. LST was highest in summer, lower in spring and autumn, and lowest in winter, regardless of diurnal differences.

**Figure 4:**
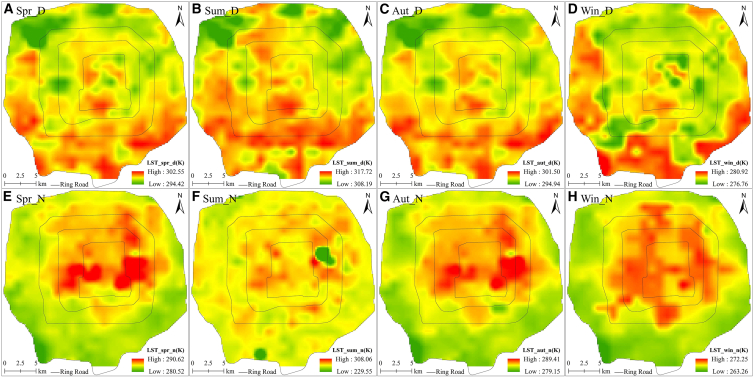
Spatial distributions of diurnal LST (A), (B), (C), and (D) represent the daytime LST in spring, summer, autumn, and winter, respectively. (E), (F), (G), and (H) represent the nighttime LST in spring, summer, autumn, and winter, respectively.

The spatial distribution of LST varied markedly between daytime and nighttime (Figure 4). Daytime LST was markedly lower in the northern half than in the southern half of each zone. This is potentially because the amount of vegetation and number of water bodies in the northern half of each zone (for instance, the Summer Palace, Old Summer Palace, Kunming Lake, Fuhai, and Yangshan River) are increased compared with those in the southern half. During the daytime, vegetation exerts a significant shading effect and enhanced transpiration, thereby providing effective cooling; conversely, water bodies warm slowly owing to their high specific heat capacity. During daytime, surface albedo is low, especially that of artificial surfaces, resulting in increased absorbed solar radiation and rapid temperature rise; these factors may explain why the conditions in the northern areas, which have more vegetation, were cooler than those in the southern areas. Nighttime LST was lower at the periphery and higher toward the center, potentially because of the absence of solar radiation and enhanced heat dissipation during nighttime. The Fifth Ring Road area contains parks and leisure areas (such as the Summer Palace, Old Summer Palace, Liangshan Park, Linglong Park, Chaoyang Park, and the International Golf Course) with an increased amount of vegetation and a low proportion of impervious surfaces; therefore, the amount of heat absorbed in this zone is reduced during daytime. The Fourth Ring Road zone contains a lower amount of vegetation and a higher proportion of impervious surfaces; therefore, the absorbed solar radiation here during daytime is increased compared with that in the Fifth Ring Road zone. Consequently, the Fifth Ring Road zone exhibited decreased sensible heat flux and increased latent heat flux compared with those in the other zones, causing LST at the periphery of Beijing to be lower than that in the middle (especially in Zone 2 and Zone 3). Based on the above analysis, it can be observed that a distinct concentric LST gradient emerges from the second to the fifth loop, closely tied to the evolutionary history of the loop morphology. However, for summer nights, there was no clear pattern of higher temperatures at the northern and southern ends of the zone. This may be because, in summer, the amount of heat absorbed by water during daytime is greater than that absorbed during nighttime, while, because of its greater specific heat capacity, water releases heat more slowly than other surfaces. In summer, air humidity, which is coupled to temperature,^57^ is higher during nighttime than during daytime. For summer nights, the temperature drop along the periphery of the study area was more gradual than that in the middle, thereby reducing the difference in surface temperature between the middle and the periphery; consequently, the temperatures in the middle were not distinctly higher than those in the north and south.

### Spatial distributions of UMPs

**Figure 5:**
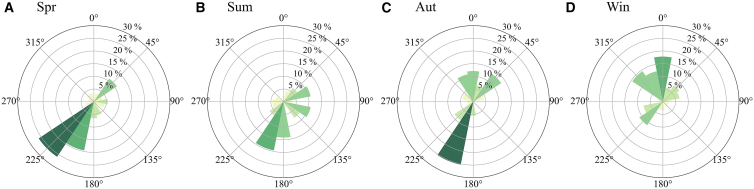
Rose diagrams of wind directions (A), (B), (C), and (D) represent the results in spring, summer, autumn, and winter, respectively.

Wind direction is required for the FAI calculations. The frequency of the 16 wind directions in each season was calculated (Figure 5) based on the daily meteorological monitoring data. The wind direction with the highest frequency was selected, and its corresponding angle was included in the FAI calculations. The wind direction angles used to calculate FAI in spring, summer, autumn, and winter were 225°, 202.5°, 202.5°, and 0°, respectively. Because the prevailing seasonal wind direction in Beijing is largely controlled by the East Asian monsoon and remains highly consistent across the urban area, the dominant wind direction used in the FAI calculation can be regarded as spatially representative of the background airflow over the study area.

**Figure 6:**
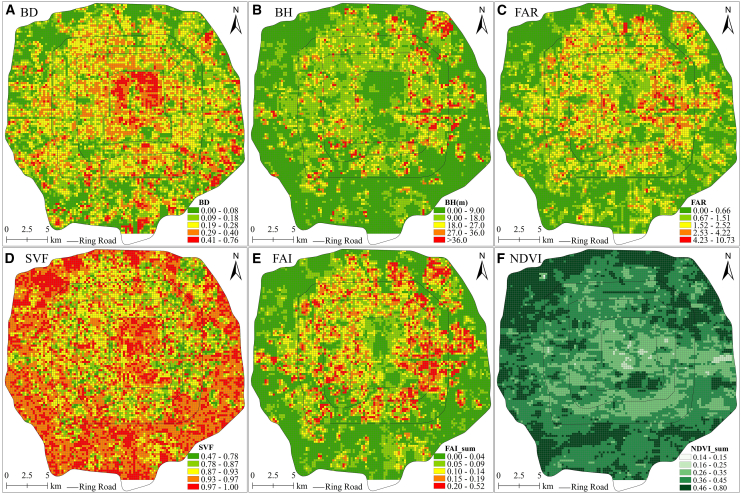
Spatial distributions of urban morphology parameters (UMPs) (A) to (F) represent the spatial distributions of BD, BH, FAR, SVF, FAI, and NDVI, respectively.

In some cases, the spatial distributions of the UMPs varied notably (Figure 6; Table S3). BH, FAR, and FAI exhibited highly similar distributions, first increasing and then decreasing from the center to the periphery. (Since the characteristics of the spatial distribution of FAI in different seasons are highly similar, only summer FAI is analyzed as an example.) This is because buildings in Zone 2 are low-rise and densely distributed, those in Zone 3 are high-rise, and those in Zone 4 tend to be low-rise. BH, FAR, and FAI are affected by building height; hence, they increased and then declined toward the periphery. BD declined from the center to the periphery, exhibiting a relatively discrete distribution. This is because buildings in Zone 2 are densely distributed, whereas those in Zone 3 and Zone 4 are relatively scattered, appearing densely distributed only in specific areas, such as commercial centers and suburban villages. SVF first decreased and then increased toward the periphery, exhibiting a more discrete distribution than that of BD. This is because buildings in Zone 2 and Zone 4 are low-rise; therefore, they cause little occlusion of the sky, leading to a large SVF. Conversely, in Zone 3, the high-rise buildings substantially occlude the sky, leading to a low SVF.

In each season, NDVI increased toward the periphery. (Only the spatial distribution of NDVI in summer is shown in the figure due to the high similarity in the spatial distribution characteristics of NDVI across seasons.) In spring, summer, and autumn, NDVI was higher in Zone 4, particularly in the upper half (Figure 6F; Table S4). This may be because Zone 4 is a recreational area with several parks (such as Liangshan Park, the Summer Palace, Old Summer Palace, and the Olympic Forest Park) and other recreational places (such as golf courses). Much of the vegetation in these areas is evergreen, causing NDVI to be high year-round. However, NDVI exhibited different distributions in each region, being lowest in Zone 2 (other than in parks) and higher in Zone 3, especially in the lower half (although more so in winter and spring than in summer and autumn). The distribution of the NDVI values differed among the seasons: Those in summer were the highest (i.e., 0.14–0.80), followed by those in autumn (i.e., 0.00–0.73), spring (i.e., 0.00–0.57), and winter (i.e., 0.00–0.47). This is primarily because vegetation exhibits substantially different distributions across the four zones, with the growth cycle of each vegetation type responding differently to seasonal changes.

### Model diagnostics and multi-scale evaluation

**Figure 7:**
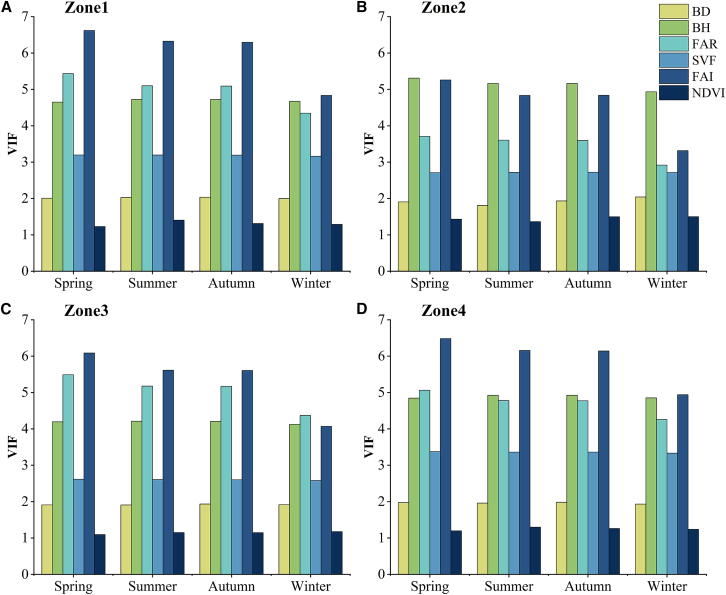
Multicollinearity test for the independent variables of the geographically weighted regression (GWR) model (A), (B), (C), and (D) represent the results in Zone 1, Zone 2, Zone 3, and Zone 4, respectively.

The variance inflation factor (VIF) was calculated to examine multicollinearity among the independent variables used in the GWR, while comparing the zones (Figure 7). For all independent variables in all zones, VIF was <7.5, indicating the absence of notable collinearity among them.^18^ Therefore, we included all the variables tested for collinearity in the GWR model.

**Figure 8:**
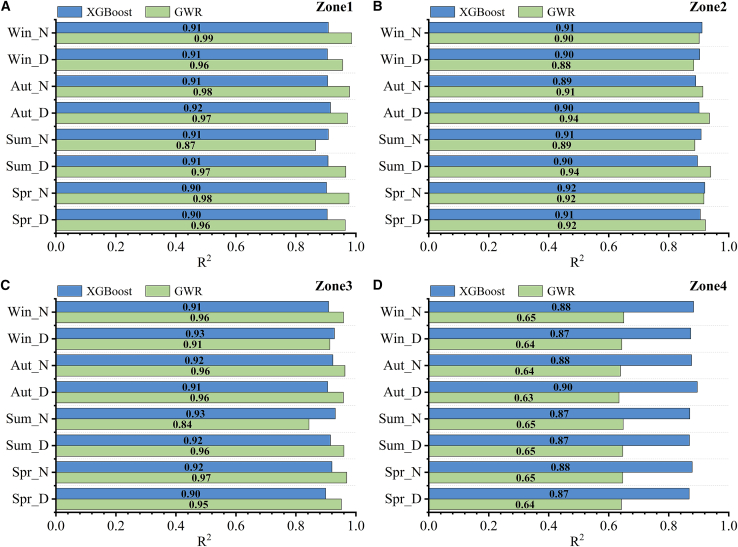
Comparison of diurnal R^2^ between the GWR and XGBoost models in different seasons (A), (B), (C), and (D) represent the results of Zone1, Zone2, Zone3, and Zone4, respectively.

To clarify the modeling hierarchy, XGBoost is treated as the primary explanatory framework to capture nonlinear and interaction effects, while OLS and GWR are employed as baseline and diagnostic references to evaluate global linearity and spatial non-stationarity, respectively. The GWR model was calibrated using a Gaussian kernel with an adaptive bandwidth. The optimal bandwidth was selected by minimizing the corrected AICc, following standard GWR calibration procedures. Additional model parameters are provided in Table S5. The ratio of the training set to the test set for the XGBoost model was 8:2.^23^^,^^58^ Conversely, since the spatial distributions of both the UMPs and the LST were characterized by strong spatial divergence, we integrated the location information (x_coord, y_coord) as a spatial feature variable to enhance the model generalization capability and prediction stability by capturing spatially autocorrelated features. Figure 8 demonstrates the coefficient of determination R^2^ of the GWR and XGBoost models for each region, with the GWR model being the result at a scale of 250 m and the XGBoost model being the result at a scale of 300 m (see Section 4.2 for details). All regression coefficients estimated by the GWR model were statistically significant (*p* < 0.05). The XGBoost model demonstrated stable and reliable predictive performance. Evidently, there is a significant nonlinear relationship between UMPs and LST,^59^ with the degree being significantly stronger than the linear relationship (see Table S6 for details regarding the OLS results). In Zone1, Zone2, and Zone3, both GWR and XGBoost perform well, with their R^2^ values being comparable. Conversely, in Zone4, XGBoost performs significantly better than GWR, which may be due to Zone4 having an increased amount of vegetation and number of water bodies, as well as less densely distributed buildings. Hence, with a decrease in the sample number, the UMP values in Zone 4 change more than those in the other zones, leading to a larger discrepancy between the R^2^ values of the two models.

### Analysis of GWR model results

**Figure 9:**
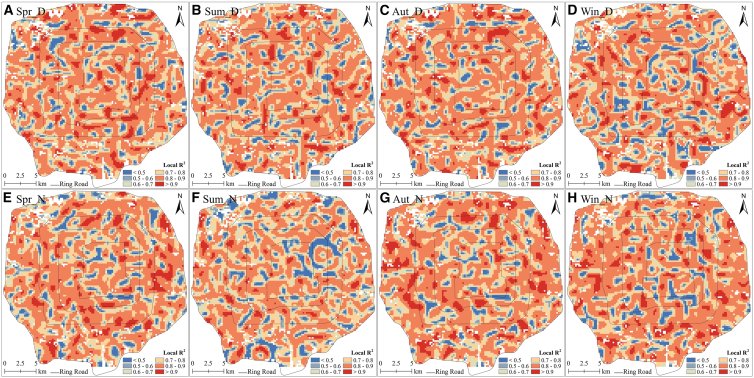
Spatial distribution of local determination coefficient (Local R^2^) of the GWR model in Zone 1 (A), (B), (C), and (D) represent daytime in spring, summer, autumn, and winter, respectively. (E), (F), (G), and (H) represent nighttime in spring, summer, autumn, and winter, respectively.

In Zone 1, the performance of the GWR model exhibited distinct spatial variation in the local determination coefficient (Local R^2^). For each season, Local R^2^ exhibited marked spatial heterogeneity and agglomeration, indicating substantial spatial non-stationarity. Traditional global regression models, such as OLS, have difficulty capturing such local changes.^54^^,^^60^ Here, Local R^2^ was mostly in the range of 0.8–0.9, indicating that at a 250-m scale, the GWR model performed well in capturing local variation in LST (Figure 9).

**Figure 10:**
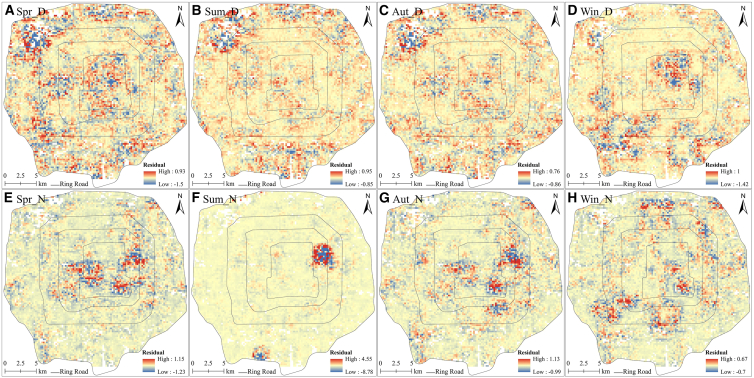
Spatial distributions of the standardized residuals of the GWR model in Zone 1 (A), (B), (C), and (D) represent daytime in spring, summer, autumn, and winter, respectively. (E), (F), (G), and (H) represent nighttime in spring, summer, autumn, and winter, respectively.

Analysis of standardized residuals can effectively reveal whether the GWR model overestimates or underestimates LST. The size of the standardized residuals reflects the accuracy of the model: The smaller the absolute value of the standardized residuals, the greater the model's accuracy. If the absolute value of a standardized residual is > 2.5, the area may contain outliers or the model may be inaccurate.^54^^,^^60^ We examined the spatial distribution of the standardized residuals of the GWR for Zone 1 (Figure 10). Considering both seasonal and diurnal variations, the standardized residuals revealed spatial nonstationarity with spatial agglomeration and distinct spatial heterogeneity. The standardized residuals exhibited greater spatial clustering during nighttime than during daytime, particularly in summer. The GWR predictions of LST were either overestimated or underestimated at each temporal scale; however, except for the summer nighttime predictions, they were within the model’s allowed range (the absolute value of standardized residuals was <2.5). For the summer nighttime, two areas (the color is darker in Figure 10F) exhibited extreme standardized residuals, having LST values significantly lower than those of the other areas (Figure 4F). This may be because the conditions in these two areas were influenced by a nearby park, generating outliers. The GWR model exhibited substantial spatial nonstationarity, effectively capturing the local variation in LST, while producing accurate estimates. Therefore, GWR was more suitable than OLS for examining the factors influencing the urban surface thermal environment.

### Impact of UMPs on LST changes

**Figure 11:**
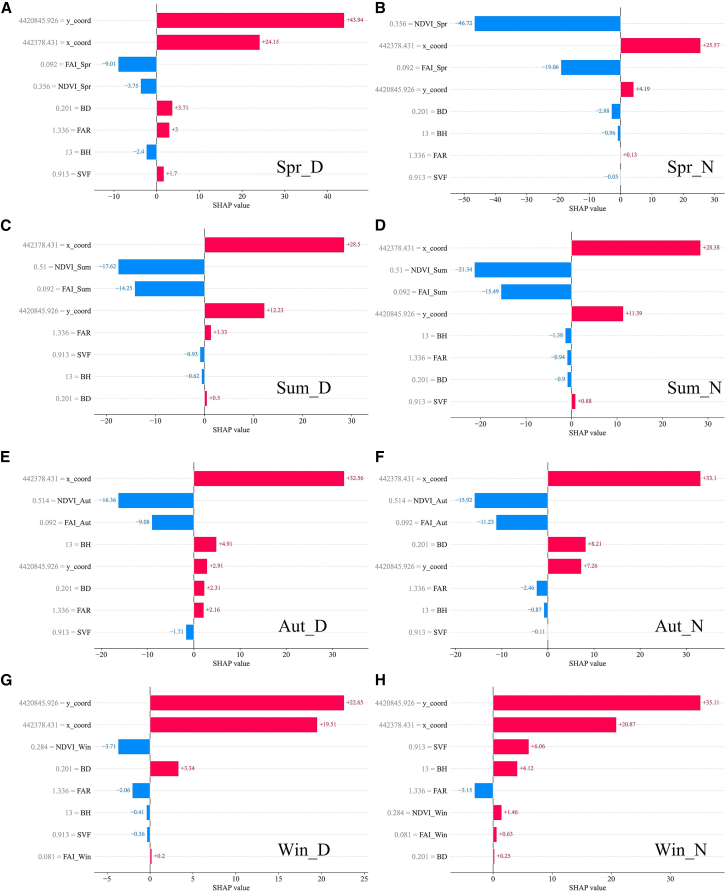
Order of local SHAP values for model predictors (A), (C), (E), and (G) represent daytime in spring, summer, autumn, and winter, respectively. (B), (D), (F), and (H) represent nighttime in spring, summer, autumn, and winter, respectively.

In this study, the SHAP model was used to decompose the effect of urban form on thermal environmental change based on the XGBoost model at a scale of 300 m. The SHAP local analysis demonstrated clearly the order and direction of the strength of the effect of urban form on thermal environmental change (positive values indicate positive driving, whereas negative values indicate negative regulation) (Figure 11). Among all variables, the spatial coordinates x_coord and y_coord showed relatively strong positive contributions. These substantial SHAP magnitudes quantify the strength of the underlying spatial gradient and indicate that location accounts for considerable background variability in LST patterns. This pattern does not imply that geographical location directly drives thermal change; rather, these variables capture the large-scale spatial gradient of Beijing’s thermal field—from the cooler mountainous northwest to the warmer, densely built southeastern plain. Their high SHAP values, therefore, reflect the underlying spatial structure of the city and the strong spatial autocorrelation inherent in the urban thermal environment.^61^ Overall, NDVI and FAI exerted strong negative effects on LST in spring, summer, and autumn, as indicated by their generally negative SHAP contributions across these seasons. In winter, the negative effect of NDVI on LST weakened markedly, remaining negative during daytime (−3.71) but shifting to a slight positive contribution at nighttime (+1.46), whereas FAI showed weak positive effects in both daytime and nighttime. The weaker inhibitory effect of NDVI on LST change in winter compared with the cases in the other seasons is closely related to the growth status of vegetation in winter. FAI is an urban morphology indicator that is closely related to the wind environment, inhibiting the LST increase in spring, summer, and autumn, which implies the significant role of natural ventilation in mitigating the heat island effect. BD has a positive promoting effect on the thermal environmental change as a whole; however, it is weaker compared with those of NDVI and FAI. Densely distributed buildings absorb a large amount of solar radiation while simultaneously hindering ventilation. These two effects combined contribute to the LST increase.

**Figure 12:**
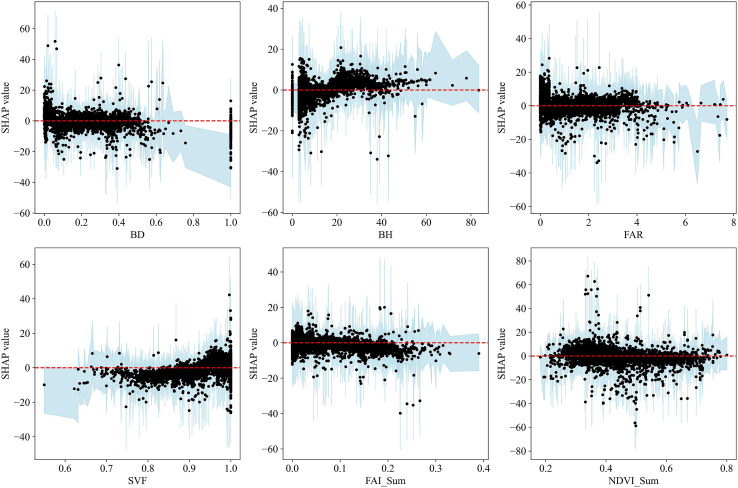
Partial dependence plots of the SHAP values for UMPs during summer daytime Black dots represent bootstrap-averaged SHAP values at the observed predictor values. The light-blue shaded band indicates the 95% bootstrap confidence interval (2.5th–97.5th percentiles), and the red dashed line marks SHAP = 0.

Although the SHAP partial analysis can show the strength and direction of the effect of UMPs on the changes of the thermal environment, it does not portray the trend of the effect of the increasing values of UMPs on the LST changes; hence, we further plotted the partial dependence of SHAP for each UMP (Figure 12). We note that since the trends of SHAP values of UMPs with their values are highly similar in each time period, we analyzed only the results during daytime in summer. According to the results, as the values of BD, FAR, SVF, FAI, and NDVI increase, the respective SHAP values remain relatively stable—i.e., the degree of influence on LST changes does not vary with their values. As the value of BH increases, its SHAP value gradually increases, the sign changes from negative to positive, and the threshold value (the change in the sign of the SHAP value—i.e., the value of the change in the direction of action) is about 20 m—i.e., the promotion of the LST increase is gradually increasing. This indicates a nonlinear threshold beyond which BH tends to exert an increasingly positive effect on LST. To quantify uncertainty, we estimated 95% bootstrap confidence intervals (2.5th–97.5th percentiles) for the SHAP partial dependence curves, and the BH threshold consistently remains centered around ∼20 m across resamples. On the one hand, with an increase in BH, the surface area of the building is increasing and the absorbed solar radiation is increasing; on the other hand, an increase in BH will hinder the heat dissipation effect of natural ventilation. Hence, future urban planning should consider the influence of building height on the changes of the thermal environment.^61^^,^^62^

**Figure 13:**
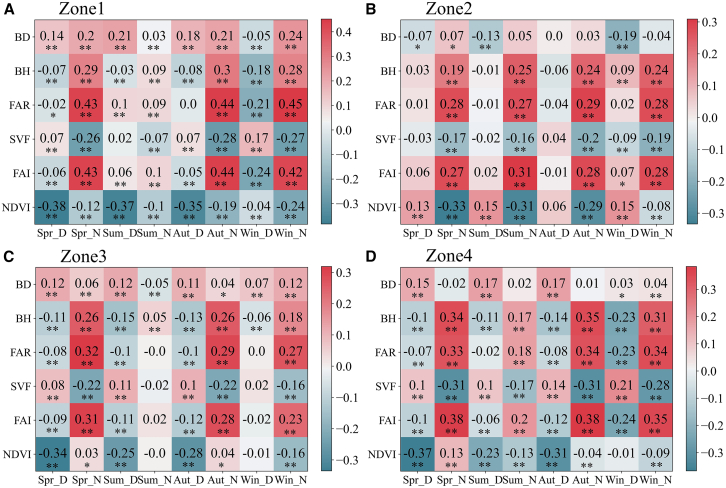
Correlation of UMPs with diurnal LST (A), (B), (C), and (D) represent the results in Zone1, Zone2, Zone3, and Zone4, respectively. Spr_D, Spr_N, Sum_D, Sum_N, Aut_D, Aut_N, Win_D, and Win_N represent spring daytime, spring nighttime, summer daytime, summer nighttime, autumn daytime, autumn nighttime, winter daytime, and winter nighttime, respectively. One asterisk indicates significant correlation at the 0.05 level, and two asterisks indicate significant correlation at the 0.01 level, based on Pearson’s correlation analysis.

The correlations varied among the zones. Overall, LST was more strongly correlated with the 3D UMPs than with the 2D UMPs,^63^ and UMPs were more strongly correlated with nighttime LST than with daytime LST (Figure 13). In Zone 1, BD was generally positively correlated with LST, and the correlation was stronger during nighttime than during daytime. BH was strongly positively correlated with nighttime LST in winter, spring, and autumn. FAR and FAI were similarly correlated with LST, with strong positive nighttime correlations in winter, spring, and autumn and negative daytime correlations in winter. SVF was strongly negatively correlated with nighttime LST in spring, autumn, and winter and positively correlated with daytime LST in winter. NDVI was negatively correlated with LST, more strongly during daytime than nighttime, in spring, summer, and autumn; however, this effect was reversed in winter. In Zone 2, BD showed negative correlations with daytime LST in spring, summer, and winter. BH, FAR, and FAI exhibited similar correlations with LST and stronger positive correlations with LST during nighttime than during daytime. SVF was negatively correlated with nighttime LST. NDVI was strongly negatively correlated with nighttime LST in spring, summer, and autumn. In Zone 3, BD was positively correlated with daytime LST in spring, summer, and autumn and with nighttime LST in winter. BH, FAR, and FAI exhibited similar correlations with LST and stronger positive correlations with nighttime LST in winter, spring, and autumn. SVF was negatively correlated with nighttime LST in winter, spring, and autumn. NDVI was strongly negatively correlated with daytime LST in spring, summer, and autumn and with nighttime LST in winter. In Zone 4, BD was positively correlated with daytime LST in spring, summer, and autumn. BH, FAR, and FAI exhibited similar correlations with LST and stronger positive correlations with nighttime LST. SVF was more strongly negatively correlated with nighttime LST than with daytime LST. NDVI exhibited stronger negative correlations with daytime LST than with nighttime LST in spring, summer, and autumn.

The effects of urban morphology and vegetation on LST differed between Zone 2 and the other zones. In Zone 2, BD weakly inhibited the increase in daytime LST in spring, summer, and winter; in the other zones, in spring, summer, and autumn, it weakly promoted the increase in daytime LST. This counterintuitive effect highlights a Beijing-specific nuance: in certain urban zones, dense low-rise buildings may create shading that mitigates daytime heating, contrary to the conventional expectation. This indicates that BD did not significantly promote an increase in daytime LST. BH, FAR, and FAI exerted the same effects on LST in Zones 1, 3, and 4: In winter, spring, and autumn, they strongly enhanced the increase in nighttime LST, whereas in Zone 2, they strongly enhanced the increase in nighttime LST in all seasons. This may be because impermeable surfaces absorb large amounts of solar radiation during daytime, whereas they primarily dissipate heat during nighttime. High-rise buildings hinder air circulation and reduce nocturnal radiative and convective heat dissipation, slowing the release of stored heat after sunset. SVF inhibited the increase in nighttime LST, as greater sky exposure enhances nocturnal radiative heat loss, whereas lower SVF indicates a stronger canyon enclosure that restricts longwave radiation escape. In Zone 2, NDVI strongly inhibited the increase in nighttime LST, whereas in other regions, it strongly inhibited the increase in daytime LST. This may be because Zone 2 has a relatively lower amount of vegetation and therefore no significant transpiration effect, whereas in the other areas, which have relatively higher amounts of vegetation, the transpiration effect is significant. Urban morphology (in terms of the building pattern) and vegetation (in terms of its distribution) affected the thermal environment in Zone 2 more strongly, involving more complex effects, compared with the cases in the other zones.

**Figure 14:**
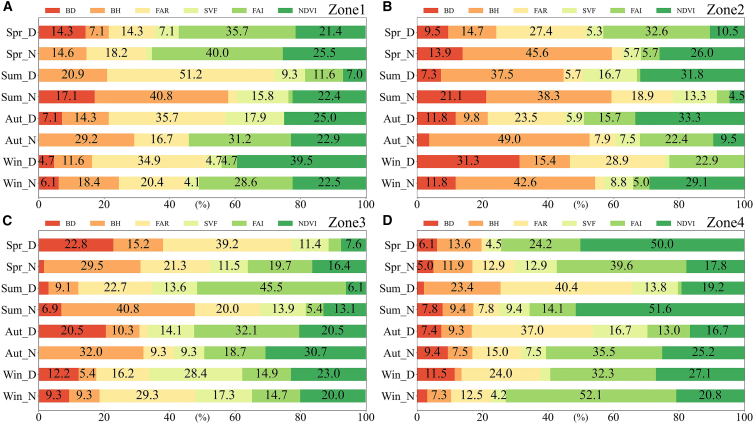
Effects of UMPs on diurnal LST changes (A), (B), (C), and (D) represent the results in Zone 1, Zone 2, Zone 3, and Zone 4, respectively. Spr_D, Spr_N, Sum_D, Sum_N, Aut_D, Aut_N, Win_D, and Win_N represent spring daytime, spring nighttime, summer daytime, summer nighttime, autumn daytime, autumn nighttime, winter daytime, and winter nighttime, respectively.

The proportions of diurnal LST variation explained by each of the drivers varied substantially (Figure 14). In Zone 1, BH affected nighttime LST more in summer and autumn than in winter and spring, explaining 20–40% of the LST variation. FAR explained 20–50% of the variation in daytime LST in summer and autumn, as well as in daytime and nighttime LST in winter. FAI contributed 28–40% to nighttime LST in spring, autumn, and winter. NDVI contributed >20% to LST in autumn, winter, and spring. BH, FAR, FAI, and NDVI contributed more than the other parameters to LST, possibly because (1) plant transpiration alleviates the daytime LST increase; (2) during nighttime, in the absence of solar radiation, surface heat dissipates; and (3) high-rise buildings do not support air circulation and thus promote an LST increase. In Zone 2, BD contributed >20% to nighttime LST in summer and daytime LST in winter. BH contributed 38–50% to nighttime LST. FAR exerted a larger effect on daytime LST in spring, autumn, and winter (contributing ca. 30%) compared with that in summer. FAI contributed 20–32% to daytime LST in spring, nighttime LST in autumn, and daytime LST in winter. NDVI contributed 26–34% to nighttime LST in spring, daytime LST in summer and autumn, and nighttime LST in winter. In Zone 2, the drivers affected the daytime LST variation more than the nighttime LST variation, possibly because of the reduced amount of vegetation, particularly between buildings, resulting in more absorbed heat, promoting an LST increase. In Zone 3, BD contributed ca. 20% to daytime LST in spring and autumn. BH contributed 29–41% to nighttime LST in spring, summer, and autumn. FAR contributed 20–40% to LST in spring, summer, and winter. SVF contributed ca. 20% to LST in winter. FAI contributed 18–46% to LST in several spring, summer, and autumn scenarios. NDVI contributed more to LST in autumn and winter (i.e., 20–31%) than to LST in spring and summer. All of these factors exerted a marked effect on the LST variation in this zone, and the height-related indicators exhibited greater contributions, which may be because the buildings in this zone are tall, albeit sparsely distributed. NDVI contributed more to LST in autumn and winter than to LST in spring and summer, possibly owing to the high proportion of evergreen vegetation in this region. In Zone 4, FAR contributed 24–41% to daytime LST in summer, autumn, and winter. FAI contributed 13–53% to LST in spring, autumn, and winter. NDVI contributed ca. 20% to LST; however, it contributed ≥50% during daytime in spring and nighttime in summer. In this zone, FAR and FAI affected the LST variation more than the other UMPs, possibly because the buildings are more sparsely distributed compared with the cases in the other zones, while this zone exhibits strong urban-canyon convection. In some of the seasons, FAI and NDVI contributed more to LST than the other UMPs. This reflects that, in some places, the urban wind environment and vegetation significantly affect the LST variation.

The LST variation was affected more by the 3D UMPs than by the 2D UMPs. BD (a 2D UMP) contributed substantially to LST in Zone 2. BH contributed substantially to LST in Zone 1, Zone 2, and Zone 3; in Zone 2, it contributed ca. 40% to nighttime LST. FAR and FAI contributed more to LST in Zone 1, Zone 3, and Zone 4 than to LST in Zone 2. SVF contributed more to LST in Zone 3 than to LST in the other zones. NDVI substantially affected LST in each area, especially in Zone 1 and Zone 4, reflecting the central role of vegetation in alleviating the UHI effect. The effects of drivers on the LST variation were different in different regions. These findings indicate that city planning should be tailored to local conditions in specific areas.

### Interaction effects among influencing factors

**Figure 15:**
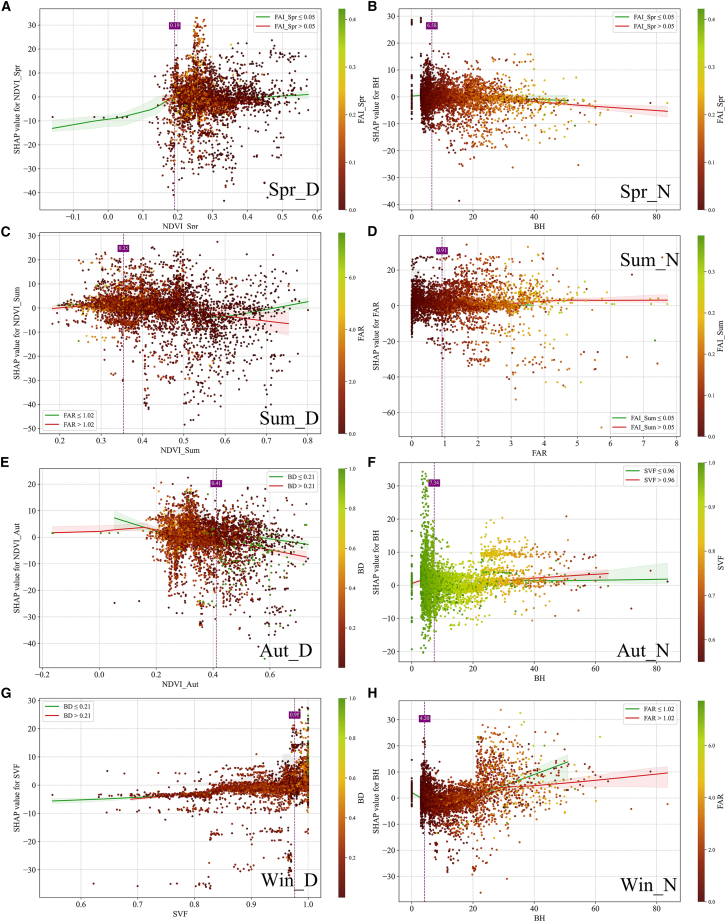
SHAP-based interaction plots of the two most influential urban morphological and vegetation-related factors under different seasonal and diurnal conditions Point colors indicate the conditioning factor. Green and red curves show LOWESS-fitted trends for below-median and above-median conditioning-factor levels, respectively; shaded bands indicate bootstrap confidence intervals, and vertical dashed lines mark approximate transition points.

To further elucidate the combined effects of urban morphology and vegetation on LST, SHAP interaction plots were employed to analyze the joint influence of major factors (Figure 15). In these interaction plots, the x axis represents the value of the primary predictor, while the y axis denotes its corresponding SHAP contribution to LST. Point colors indicate the magnitude of the conditioning factor, whereas the green and red fitted curves represent the response trends of the primary predictor under below-median and above-median levels of the conditioning factor, respectively. Vertical dashed lines mark approximate zero-crossing or transition points in the fitted SHAP response. These transition points should be interpreted as indicative thresholds derived from the SHAP interaction patterns rather than strict physical boundaries. This visualization framework facilitates a direct comparison of how the influence of a given factor varies under different morphological and ventilation conditions across seasons and diurnal periods.

The interaction results indicate that vegetation-related cooling effects are most pronounced during daytime and are strongly constrained by urban morphological conditions. During spring daytime (Spr_D), NDVI_Spr exhibits a threshold behavior around 0.19, with its cooling effect being more evident under lower FAI_Spr values, corresponding to better ventilation conditions, and substantially weakened when FAI_Spr is high. During summer daytime (Sum_D), the cooling effect of NDVI_Sum beyond approximately 0.35 is significantly modulated by FAR, with clear divergence in the cooling contribution of vegetation between different FAR conditions. A similar pattern is observed during autumn daytime (Aut_D), where NDVI_Aut shows a threshold near 0.41; its cooling effect differs between lower and higher BD conditions, indicating that the contribution of vegetation is also constrained by BD. These results consistently demonstrate that vegetation-induced cooling is highly dependent on surrounding urban form. Similar nonlinear interaction patterns between green space and 2D/3D building morphology have been observed in Xi’an, where the cooling contribution of vegetation varies with building coverage and volumetric configuration.^64^

In contrast, nighttime LST interactions are predominantly governed by three-dimensional urban morphology and are closely linked to ventilation and spatial enclosure. During spring nighttime (Spr_N), the effect of BH changes markedly beyond approximately 6.58 m, with clear differences observed between lower and higher FAI_Spr conditions. During summer nighttime (Sum_N), FAR exhibits a weak response, with its SHAP contribution remaining relatively flat across different FAI_Sum levels, despite a transition occurring around 0.91. During autumn nighttime (Aut_N), the warming contribution of BH increases markedly beyond approximately 7.34 m under higher SVF conditions, whereas during winter nighttime (Win_N), BH shows a stronger positive contribution once it exceeds approximately 4.20 m, with evident divergence between different FAR conditions. During winter daytime (Win_D), SVF shows a distinct response near 0.98, where higher SVF values enhance the absorption of low solar-angle radiation in areas with higher BD. Overall, vegetation cooling effects are primarily regulated by openness and ventilation, whereas building height, building volume, and spatial enclosure jointly control nighttime heat retention through enhanced longwave radiation trapping and reduced convective dissipation under canyon-like configurations, indicating that the urban thermal environment emerges from nonlinear interactions among multiple morphological and vegetation-related factors under different seasonal and diurnal contexts.

## Discussion

### Scale dependence and result robustness

When conducting studies on local climate and urban thermal environments, the relationship between urban morphology and LST is highly sensitive to spatial scale, a finding confirmed both in this study and in previous research.^18^^,^^32^^,^^45^^,^^56^ Based on Zone 1, multi-scale tests across nine grid sizes (100–500 m) show that GWR and XGBoost exhibit generally consistent accuracy responses to scale variation, although more pronounced fluctuations occur during summer nighttime. This likely reflects the increased complexity of driver interactions under enhanced humidity-temperature coupling, where nighttime heat storage and release and longwave radiative exchange processes jointly intensify nonlinear responses.^57^ Considering the relatively coarse resolution of the LST dataset and the need to balance downscaling error against morphological information integrity, we adopt 250–300 m as the principal explanatory scale for subsequent mechanistic analyses, corresponding to 250 m for GWR and 300 m for XGBoost.^18^

Previous studies likewise demonstrate that there is no universal optimal scale for urban morphology-thermal environment relationships; instead, the optimal scale varies with indicator attributes and spatial structure.^65^ For example, three-dimensional metrics such as FAR and mean building height tend to be more sensitive at intermediate scales, whereas land-use proportion indicators often gain explanatory power at broader scales, suggesting structural scale shifts associated with spatial aggregation.^66^^,^^67^ Multi-scale regression analyses further indicate that, in high-density cores, street-canyon ventilation and enclosure effects are detectable primarily at finer scales,^68^ while in open communities or green-space systems, larger scales are required to capture corridor-based synergistic cooling.^69^ Similarly, vegetation-related indicators exhibit marked scale dependence: street-level studies show that the cooling effect of NDVI varies with buffer radius, peaking at approximately 40 m and stabilizing at larger scales.^70^ When the scale is too fine, limited within-unit samples and excessive morphological heterogeneity may destabilize relationships; conversely, overly coarse scales may obscure three-dimensional morphological signals. Thus, the “optimal scale” identified in different cities fundamentally reflects the matching between spatial organizational hierarchy and energy exchange processes. In comparison, Beijing’s ring-road-cluster structure provides relatively stable spatial units, making the 250–300 m range more likely to capture a stable morphology-thermal coupling. This concentrated scale interval is therefore not primarily driven by model differences, but by the joint effects of urban structural hierarchy, seasonal and diurnal energy-dominant mechanisms, and the indicator system.

After clarifying the scale structure, it is necessary to assess the robustness of results across modeling frameworks. While the multi-model framework enhances explanatory capacity, it also introduces methodological uncertainty arising from differences in model assumptions and calibration strategies. OLS serves as a global linear reference, GWR reveals spatial heterogeneity, and XGBoost with SHAP identifies nonlinear relationships and variable interactions; therefore, differences arise in estimated effect magnitudes and threshold ranges. Such uncertainty mainly concerns variations in contribution intensity and local expression, rather than changes in effect direction or scale structure. Notably, GWR and XGBoost exhibit consistent scale-response trends, and the key diurnal findings remain structurally stable: three-dimensional morphological indicators exert stronger warming effects on nighttime LST, whereas NDVI shows more pronounced negative correlations with daytime LST in spring, summer, and autumn, with weakened effects at night. Hence, model differences affect explanatory detail but do not alter the overall judgment regarding urban thermal response mechanisms and their intermediate-scale coupling characteristics. The consistency of these findings across models underscores the robustness of our methodological framework, highlighting that careful spatial delineation is essential for capturing reliable urban thermal patterns.^71^

### Physical mechanisms of urban morphological effects and diurnal differences

The surface thermal environment is governed by the surface energy balance. Urban morphology regulates heat storage and release by altering spatial enclosure, ventilation conditions, and radiative exchange pathways.^72^ Compared with two-dimensional indicators, 3D UMPs are more directly involved in these energy pathways and therefore exhibit stronger associations in this study, particularly during nighttime. Their effects are related to mechanisms such as the urban canyon effect,^73^ multiple radiative reflections,^74^ and ventilation resistance,^75^^,^^76^ which reshape the nocturnal surface energy balance by modifying longwave radiation retention and sensible heat release rhythms. The regression results across zones further clarify these mechanisms. Zone 2 is characterized by low-rise but densely distributed buildings, high enclosure, and restricted ventilation; accordingly, nighttime LST shows consistently enhanced responses to BH, FAR, and FAI. Zone 3 consists mainly of high-rise yet relatively dispersed buildings; height-related indicators still dominate nighttime responses, but their spatial heterogeneity produces stronger localized variability. In Zone 4, where vegetation and water bodies are more abundant, the regulatory role of NDVI becomes more prominent. These regional differences suggest that the impact of 3D morphology on the thermal environment is not a simple linear amplification, but a structural effect jointly shaped by building volume, layout density, and underlying surface composition, indicating that morphological effects are embedded within specific spatial configurations rather than representing independent additive influences.^77^

The transferability of these mechanisms is supported by findings from other cities. In Wuhan, multi-scale analyses show that 3D building morphology indicators explain LST significantly better than 2D indicators and exhibit pronounced nonlinear enhancement,^64^ consistent in direction with the nighttime dominance of 3D morphology observed in Zones 2 and 3. Studies in European cities based on local climate zone classifications similarly report stronger nighttime heat retention in compact built types, while open or naturally covered types maintain lower temperatures.^66^ This diurnal contrast aligns with our findings that 3D morphological effects intensify at night, whereas vegetation regulation is more pronounced during the daytime. However, unlike Wuhan or Xi’an, which are characterized by single-core structures or basin topography,^67^ Beijing’s ring-road-based multi-ring spatial organization strengthens the coupling between morphological gradients and thermal-field gradients, leading to clearer zonal differentiation in thermal responses. This multi-ring structure amplifies nighttime heat accumulation contrasts across spatial units, thereby enhancing the zonal differentiation of 3D morphological effects.

From diurnal and seasonal perspectives, the regulatory pathways of urban morphology and vegetation also shift markedly. BH, FAR, and FAI show significantly stronger positive associations with nighttime LST, while during daytime, they exhibit weak correlations or localized suppression. In contrast, NDVI exerts a stable cooling effect during daytime but a weakened influence at night. This contrast reflects a shift in dominant energy processes from “shortwave radiation absorption-latent heat exchange” during daytime to “heat storage release-longwave radiation regulation” at night. During the day, shortwave absorption and evapotranspiration dominate, and vegetation effectively reduces LST through shading and latent heat exchange.^78^ At night, in the absence of solar radiation, heat storage release and longwave radiation exchange prevail. High-rise and high-FAR buildings reduce sky-view factor, enhance multiple radiative reflections, and restrict convective ventilation, thereby limiting effective longwave radiation escape and prolonging radiative retention within urban canyons, ultimately intensifying nighttime heat retention. The stronger negative correlation of SVF with nighttime LST further underscores the importance of radiative dissipation conditions for nocturnal cooling. The relatively weakened morphological effects during summer nighttime may be associated with enhanced humidity-temperature coupling and more complex latent heat exchange processes.^57^ Under humid conditions, an increased proportion of latent heat flux may reduce the dominance of sensible heat storage and release in controlling nighttime LST, suggesting that the morphological regulation of nocturnal heat release becomes more diversified in high-humidity contexts.

### Factor interactions and contextual dependence

Interaction analysis indicates that the effects of urban morphology and vegetation on LST do not operate independently; rather, they exhibit structurally dependent coupling under specific seasonal and diurnal contexts. The cooling effect of vegetation is not an intrinsic and fixed property, but is constrained by surrounding building morphology and ventilation conditions. Although NDVI generally contributes to daytime cooling, its marginal effect is substantially constrained in areas characterized by high BD, large building volume, or elevated ventilation resistance. Under such conditions, restricted air circulation limits the spatial diffusion of evapotranspiration-induced cooling, thereby reducing the effectiveness of vegetation in regulating daytime LST.^79^ This suggests that whether daytime vegetation effects can be effectively realized depends on whether the morphological configuration provides sufficient conditions for air diffusion and energy exchange. In contrast, the interaction logic at night follows a different mechanism. Compared with the daytime dominance of latent heat exchange, nighttime LST is more strongly regulated by heat storage release and longwave radiation processes. In this study, the overall explanatory power of NDVI weakens at night, and its marginal effect is primarily conditioned by enclosure structure and ventilation. In highly enclosed or poorly ventilated areas, heat retention and delayed release within the built environment weaken or even offset vegetation effects, resulting in weak synergy or antagonism. Conversely, in spatial units with lower enclosure and better ventilation, vegetation cooling more readily couples with air exchange, forming a relatively stable synergistic relationship.^80^^,^^81^ This diurnal shift in dominant energy structure is consistent with the SHAP interaction patterns observed across different Zones and ventilation contexts. Moreover, such conditional synergy or antagonism is more pronounced during daytime in the warm season, whereas in the cold season and at night, three-dimensional built morphology more strongly governs LST responses, highlighting the temporal dependence of interaction mechanisms.

Overall, this study reveals conditional synergy and constraint relationships between built morphology and vegetation under different energy-dominant phases, rather than simple linear superposition. By situating these interaction boundaries within a unified seasonal-diurnal and multi-scale framework, it moves beyond previous studies that focus on single temporal scales or examine built and vegetated factors separately. From a mechanistic perspective, this integrated approach explains the pronounced seasonal and spatial heterogeneity of LST patterns and provides a structural basis for differentiated and scale-sensitive heat-mitigation strategies.

### Implications and recommendations

With rapid urbanization, urban UHIs are gradually becoming more common, having potentially detrimental effects on local residents.^32^^,^^54^ Based on the diurnal and seasonal dominant mechanisms identified in this study, heat mitigation should not rely solely on greening or density control. Instead, a coordinated framework integrating UMPs, ventilation structure, and vegetation configuration is required. Given Beijing’s ring-road-based, concentric high-density structure, clusters within the second to fourth ring roads, particularly compact blocks characterized by high FAR and low SVF, are more prone to forming nighttime heat-retention units. In built-up area renewal, introducing stepped building-height transitions and increasing inter-building spacing while avoiding continuous high-rise enclosure interfaces can enhance street-level ventilation connectivity. In new or redevelopment projects, preserving ventilation corridors aligned with prevailing wind directions is essential to improve nocturnal heat dissipation. In terms of vegetation, greening efforts should prioritize central areas characterized by “high density-low NDVI,” especially where high FAR suppresses vegetation effectiveness. Under Beijing’s land constraints, rooftop greening, vertical greening, and pocket green spaces offer feasible solutions. In large public buildings and old residential renewal, vegetation increment and structural optimization should be implemented simultaneously to improve the spatial balance of green coverage and strengthen daytime cooling during the warm season. Amid increasingly frequent extreme heat events,^82^ the combined optimization of UMPs and three-dimensional greening is urgently needed to reduce sustained heat exposure, particularly in high-density. Planning responses can therefore be summarized as controlling excessive enclosure, improving ventilation continuity, and enhancing vegetation coverage. To enhance policy operability, the proposed pathway of “controlling enclosure—ensuring ventilation—promoting vertical greening” can be translated into graded control units and renewal guidelines, linking heat-risk identification with planning regulation.^83^^,^^84^ Meanwhile, embedding heat considerations into comprehensive and renewal planning systems, supported by cross-sector coordination, is essential for strengthening long-term UHI governance.^85^

### Limitations of the study

First, the low spatial resolution LST data were resampled to obtain higher resolution data. In the future, higher resolution LST data, such as ECOSTRESS LST data, will become available and should provide improved estimates.^86^^,^^87^^,^^88^ Second, we considered only the impacts of urban morphology and vegetation on the thermal environment. In the future, factors such as the characteristics of impermeable surfaces and water bodies should also be included. In addition, this study focused only on Beijing, which exhibits relatively typical UHI effects. However, in the case of other cities, the impacts of other drivers should be thoroughly examined. Finally, only three models, namely OLS, GWR, and XGBoost, were used in this study, and more machine learning algorithms, such as Random Forest, Boosted Regression Tree, and Light-GBM, should be considered in future studies.

## Resource availability

### Lead contact

Requests for further information and resources should be directed to and will be fulfilled by the lead contact, Junling Jin (junling.jin@gxnu.edu.cn).

### Materials availability

This study did not generate new unique reagents.

### Data and code availability

• Data: Building vector data for Beijing were obtained from the Resources and Environment Science Data Platform (RESDC) and are available at https://www.resdc.cn/data.aspx?DATAID=270, generated by integrating Baidu Maps data with LiDAR and high-resolution remote sensing imagery (overall accuracy >94%). All-weather land surface temperature (LST) data at 1 km resolution were acquired from the Third Pole Environment Data Center (TPDC) at https://data.tpdc.ac.cn/zh-hans/data/05d6e569-6d4b-43c0-96aa-5584484259f0, constructed using MODIS LST products, GLDAS reanalysis data, and auxiliary variables (vegetation index, surface albedo) with good accuracy under both clear and cloudy conditions. Daily wind direction and speed observations (16 compass sectors, precision 0.1 m/s) were obtained from the China Meteorological Data Service Center (CMDC) at http://data.cma.cn. Normalized difference vegetation index (NDVI) data at 250 m resolution were derived from the MOD13Q1 product and provided by TPDC at https://data.tpdc.ac.cn/zh-han/data/f3bae344-9d4b-4df6-82a0-81499c0f90f7, processed via Savitzky-Golay filtering and maximum value compositing. All datasets were resampled to 100–500 m grids for spatial consistency. The code used for data processing and analysis is available from the corresponding author upon reasonable request.
• Code: Custom scripts used for data processing and analysis are available from the lead contact upon reasonable request.
• Additional information: Any additional information required to reanalyze the data reported in this paper is available from the lead contact upon request.

## Acknowledgments

This work was supported by the 10.13039/501100018571Specific Research Project of Guangxi for Research Bases and Talents (grant no. 2022AC21142), the Project on Enhancement of Basic Research Ability for Young and Middle-aged Teachers in Guangxi Universities (grant no. 2023KY0069), and the Guangxi Key Laboratory of Environmental Processes and Remediation in Ecologically Fragile Regions, Guangxi Normal University (grant no. EPRZR2024-01).

## Author contributions

H.Z.: conceptualization, resources, supervision, project administration, funding acquisition, and writing – review and editing. Y.L.: methodology, validation, investigation, writing – review and editing, and visualization. C.L.: investigation. X.S.: investigation. J.J.: conceptualization, methodology, software, validation, formal analysis, investigation, data curation, and writing – original draft.

## Declaration of interests

The authors declare no competing interests.

## Declaration of generative AI and AI-assisted technologies in the writing process

During the preparation of this work, the authors used ChatGPT to improve the clarity and readability of the manuscript. After using this tool, the authors reviewed and edited the content as needed and take full responsibility for the content of the publication.

## STAR★Methods

### Key resources table

**Table:** 

REAGENT or RESOURCE	SOURCE	IDENTIFIER
**Deposited data**		

Beijing building vector dataset	Resources and Environment Science Data Center (RESDC)	https://www.resdc.cn/data.aspx?DATAID=270
1-km all-weather land surface temperature dataset	Third Pole Environment Data Center (TPDC)	https://data.tpdc.ac.cn/zh-hans/data/05d6e569-6d4b-43c0-96aa-5584484259f0
250-m normalized difference vegetation index dataset (MOD13Q1, China, 2000–2022)	Third Pole Environment Data Center (TPDC)	https://doi.org/10.11888/Terre.tpdc.300328
Surface meteorological observations (wind direction and wind speed)	China Meteorological Data Service Center (CMDC)	http://data.cma.cn

**Software and algorithms**		

ArcGIS 10.8	Environmental Systems Research Institute, Inc	https://www.esri.com
Custom scripts for data processing and analysis	This paper	Available from the lead contact upon reasonable request

### Experimental model and study participant details

This study did not involve human participants, animals, plants, microbial strains, cell lines, or primary cell cultures. Therefore, sex- or gender-based analyses were not applicable to this study.

### Method details

#### Study area

Beijing (39°26′–41°03′ N, 115°25′–117°30′ E), the capital of China, is located in the northern North China Plain and has a warm temperate, semi-humid to semi-arid monsoon climate, characterized by hot, rainy summers and cold, dry winters. Strong seasonal contrasts in temperature and vegetation activity, driven by the East Asian monsoon, contribute to pronounced seasonal variability in the urban thermal environment. As of the end of 2024, Beijing had a permanent population of approximately 21.83 million. Rapid urbanization has intensified urban heat island (UHI) effects and produced clear gradients in building density, building height, and land-use intensity from the urban core to the outer suburbs.

The study area covered the region within the Fifth Ring Road (Zone 1; ca. 667 km^2^; Figure 1), which contains diverse building types and heterogeneous vegetation patterns. To capture intra-urban differences in morphology and greening, Zone 1 was further subdivided into the area within the Second Ring Road (Zone 2), the area between the Second and Fourth Ring Roads (Zone 3), and the area between the Fourth and Fifth Ring Roads (Zone 4). Zone 2 is mainly low-rise and densely built, Zone 3 contains many high-rise buildings, and Zone 4 is characterized by relatively sparse buildings and more vegetation and water. This zonal division supports a more precise assessment of how urban morphology and vegetation influence the thermal environment.

#### Study design and workflow

Figure 2 illustrates the research process. First, the values of UMPs were calculated at various spatial scales based on the building-vector and wind-direction data. Second, the monthly NDVI data were aggregated into seasonal data and subsequently resampled to generate data matching the scale of the UMPs. Third, the daily LST data were processed into seasonal data and subsequently resampled to match the scale of the UMP and NDVI data.^6^ Fourth, the optimal regression model and corresponding optimal scale were determined. Finally, the spatial distribution of the UMPs and NDVI, their correlation with LST, and their relative effects on thermal environmental changes were analyzed.

#### Data acquisition and preprocessing

Building vector data were obtained from the Resources and Environment Science Data Platform (RESDC), derived from Baidu Maps combined with LiDAR and high-resolution remote sensing imagery, providing building footprints and floor numbers with an overall accuracy exceeding 94%.^89^

Land surface temperature (LST) data at 1 km resolution were sourced from the Third Pole Environment Data Center (TPDC). An all-weather LST product was constructed using MODIS thermal infrared observations and GLDAS reanalysis data, integrating vegetation indices, surface albedo, and high- and low-frequency LST components. Both daytime (approximately 13:30 local time) and nighttime (approximately 01:30 local time) observations from Aqua MODIS were utilized. LST fields were resampled to analysis grids ranging from 100 to 500 m using bilinear interpolation.^90^^,^^91^

Wind direction and speed data were obtained from the China Meteorological Data Service Center (CMDC), providing daily observations across 16 compass sectors.

Normalized difference vegetation index (NDVI) data at 250 m resolution were derived from the MOD13Q1 product provided by TPDC. Noise was removed through reconstruction and Savitzky–Golay filtering, followed by monthly maximum value compositing. NDVI datasets were resampled to match the analysis grid resolution.

#### Calculation of urban morphology parameters

Six indicators were employed to characterize urban form and greening: building density (BD), building height (BH), floor area ratio (FAR), sky view factor (SVF), frontal area index (FAI), and NDVI. BD represents the proportion of building footprint area within each grid cell. BH denotes mean building height per grid. FAR reflects the ratio of total floor area to grid area. SVF quantifies hemispherical sky openness based on obstruction geometry. FAI describes wind-direction-dependent frontal building area normalized by grid area and was calculated using prevailing seasonal wind directions. All built-form parameters were computed using building vector data, while NDVI was derived from remote sensing data, and all indicators were aggregated consistently across spatial scales (Table S1).

#### Ordinary least squares and geographically weighted regression

Ordinary least squares (OLS) regression was applied to quantify global linear relationships between LST and predictor variables:^92^

$$y=\beta_{0}+\sum_{i=1}^{n}\beta_{i}x_{i}+\varepsilon$$

where *y* represents LST, *β*_0_ is the intercept, *β*_*i*_ is the coefficient of driver *x*_*i*_, *n* is the number of drivers, and *ε* is the random error. Geographically weighted regression (GWR) was used to account for spatial non-stationarity:

$$\begin{array}{c} y_{i}=\beta_{0}\left(u_{i},v_{i}\right)+\sum_{k}\beta_{k}\left(u_{i},v_{i}\right)x_{ik}+\varepsilon_{i} \end{array}$$

where *y*_*i*_ represents LST at sampling point *i*, (*u*_*i*_,*v*_*i*_) are the geographic coordinates of point *i*, *β*_0_(*u*_*i*_,*v*_*i*_) is the intercept at point *i*, *β*_*k*_(*u*_*i*_,*v*_*i*_) is the regression coefficient of driver *x*_*ik*_, *k* is the number of drivers, *x*_*ik*_ is the value of driver *x*_*k*_ at point *i*, and *ε*_*i*_ is the random error.

#### Pearson correlation analysis

Pearson’s correlation coefficient was used to examine linear relationships between LST and each predictor:

$$r=\frac{\sum \left(x-\bar{x}\right)\left(y-\bar{y}\right)}{\sqrt{\sum {\left(x-\bar{x}\right)}^{2}\sum {\left(y-\bar{y}\right)}^{2}}}$$

where *x* represents the value of the driver, *y* represents LST, $\bar{x}$ and $\bar{y}$ are the average values of *x* and *y*, respectively.

#### Extreme gradient boosting (XGBoost) modeling

XGBoost was implemented to model nonlinear relationships using ensemble regression trees with regularization:^93^

$$Obj=L\left(y,F\left(x\right)\right)+\sum_{K=1}^{K}\Omega \left(f_{k}\right)$$

where *L* is the loss function, and Ω(*f*_*k*_) is the regularization term to bound the model complexity.

The Taylor expansion is as follows:

$$Obj^{\left(t\right)}=\sum_{i=1}^{n}l\left(y_{i},y_{i}^{\left(t-1\right)}+f_{t}\left(x_{i}\right)\right)+\Omega \left(f_{t}\right)$$

The gradient is calculated and the error is updated based on the first- and second-order derivatives.

Hyperparameters were optimized via Bayesian optimization using five-fold cross-validation with root mean square error (RMSE) as the evaluation metric. Spatial coordinates were incorporated as features to improve model generalization.

#### SHAP interpretability analysis

SHapley Additive exPlanations (SHAP) were applied to quantify individual feature contributions to LST predictions based on cooperative game theory.^56^^,^^94^

$$\varphi_{i}=\sum_{S\in F\setminus \left\{i\right\}}\frac{|S|!\left(\left|F\right|-\left|S\right|-1\right)!}{|F|!}\left[f_{S\cup \left\{i\right\}}\left(x_{S\cup \left\{i\right\}}\right)-f_{S}\left(x_{S}\right)\right]$$

where *φ*_*i*_ is the SHAP value of feature *i*, which indicates the contribution of feature *i* to the model prediction, *F* is the set of all features, *S* is the subset of all features without feature *i*, *f*_*s*_(*x*_*s*_) is the predicted value of the subset of features *S*, *f*_*S*∪{*i*}_(*x*_*S*∪{*i*}_) is the predicted value of the subset of features *S* plus the predicted value of feature *i*, *S* is the size of the subset of features *S*, and *F* is the total number of all features.

SHAP values were used for feature ranking, partial dependence interpretation, and interaction analysis across seasonal and diurnal conditions.

### Quantification and statistical analysis

Multicollinearity among predictors was evaluated using VIF, with all values below 7.5 indicating the absence of notable multicollinearity. Model performance was primarily assessed using R^2^. For the XGBoost model, hyperparameters were optimized via Bayesian optimization using five-fold cross-validation with RMSE as the evaluation metric. Statistical significance of local GWR coefficients was evaluated at P < 0.05. Pearson correlation coefficients shown in Figure 13 were assessed using Pearson correlation analysis; one asterisk indicates P < 0.05 and two asterisks indicate P < 0.01. Analyses were conducted across spatial resolutions ranging from 100 to 500 m to identify optimal modeling scales.
