# Supplementary material for: Spatially heterogeneous thermal responses to urban form and greening: A multi-model analysis of seasonal and diurnal dynamics in beijing
Source: iScience. 2026 Apr 24;29(6):115843. doi: 10.1016/j.isci.2026.115843 (PMC13196141; doi:10.1016/j.isci.2026.115843)
Supplement: Document S1. Tables S1–S6 [file mmc1.pdf]

iScience, Volume *29*

Supplemental information

Spatially heterogeneous thermal responses to urban form and greening: A multi-model analysis of seasonal and diurnal dynamics in beijing

Hui Zheng, Yudi Li, Chen Li, Xian Su, and Junling Jin

Table S1. Schematic description of UMPs.

UMPs	Definitions	Formulas
BD<br>(–)<br>(0,1)	The ratio of total building footprint to unit grid area .	$\mathrm{BD} = \sum_{i=1}^{n} A_i \Big/ S \#(1)$<br>where, A_i denotes the area of the ith building in the buildings, n and S denote the number of buildings and the area of the unit grid, respectively.
BH<br>(m)<br>(0,+∞)	The vertical distance of a building from the ground to the top. In this study, it means the average height of the buildings in the unit grid .	$\mathrm{BH} = \sum_{i=1}^{n} BH_i \Big/ n \#(2)$<br>where, BH_i denotes the building height of the ith building and n denotes the number of buildings.
FAR<br>(–)<br>(0,+∞)	The ratio of building floor area to unit grid area .	$\mathrm{FAR} = \sum_{i=1}^{n} A_i F_i \Big/ S \#(3)$<br>where, A_i denotes the floor area of the ith building, F_i denotes the number of floors in the ith building, n denotes the number of buildings, and S denotes the unit grid area.
SVF<br>(–)<br>(0,1)	The ratio of the amount of radiation received (or emitted) at the Earth's surface to the amount of radiation emitted (or received) throughout the hemisphere .	$\mathrm{SVF} = 1 - \sum_{i=1}^{n} \sin^2 \beta_i \left(\frac{\alpha_i}{360°}\right) \#(4)$<br>where, α_i and β_i denote the azimuth and maximum building height sectors, respectively, and n denotes the total number of sectors in the sky hemisphere that are obscured by obstacles.
FAI<br>(–)<br>(0,+∞)	The ratio of the area of a building in a plane perpendicular to the wind direction to the area of the unit grid .	$\mathrm{FAI}(\theta) = \sum_{i} F_i(\theta) \Big/ S \#(5)$<br>where, $F_i(\theta)$ denotes the frontal area of the ith building in wind direction θ and S denotes the unit grid area.
NDVI<br>(–)<br>(−1,1)	The normalized difference vegetation index, representing planar vegetation coverage intensity within the unit grid.	where, NDVI = (NIR − RED) / (NIR + RED), and NIR and RED denote the near-infrared and red reflectance values, respectively.

Notes: where, A_i denotes the area of the ith building in the buildings, *n* and *S* denote the number of buildings and the area of the unit grid, respectively.; BH_i denotes the building height of the ith building and *n* denotes the number of buildings; F_i denotes the number of floors in the ith building, *n* denotes the number of buildings, and S denotes the unit grid area; α_i and β_i denote the azimuth and maximum building height sectors, respectively, and *n* denotes the total

number of sectors in the sky hemisphere that are obscured by obstacles; $F_i(\theta)$ denotes the frontal area of the ith building in wind direction θ and S denotes the unit grid area.

Table S2. Four-season diurnal LST data statistics for each Zone.

		Spr_D	Spr_N	Sum_D	Sum_N	Aut_D	Aut_N	Win_D	Win_N
Zone 1	Avg	300.18	284.43	314.38	298.78	299.28	282.82	279.06	267.7
	Min	294.4	280.5	305.86	229.55	293.9	278.98	276.12	262.64
	Max	302.55	290.62	317.72	308.06	301.50	289.41	281.34	272.25
Zone 2	Avg	300.36	286.13	314.96	300.18	299.55	284.29	278.88	269.68
	Min	298.3	283.90	313.38	298.29	298.18	282.69	276.76	268.28
	Max	302.08	289.72	317.25	308.06	300.91	287.49	279.91	272.25
Zone 3	Avg	300.05	285.38	314.73	299.29	299.26	283.79	278.69	269.28
	Min	296.34	282.78	309.35	229.55	296.2	280.78	277.26	266.42
	Max	301.77	290.62	317.25	308.06	300.76	289.41	280.08	271.56
Zone 4	Avg	300.31	283.87	314.52	298.69	299.38	282.31	279.19	267.05
	Min	294.42	280.52	308.19	278.63	294.94	279.15	277.39	263.26
	Max	302.55	286.85	317.72	302.33	301.50	284.97	280.92	271.18

Notes: LST data unit is thermodynamic temperature in Kelvin (abbreviated K).Spr_D, Spr_N, Sum_D, Sum_N, Aut_D, Aut_N, Win_D and Win_N denote spring day, spring night, summer day, summer night, autumn day, autumn night, winter day and winter night LST, respectively.

Table S3. Statistics on urban morphology parameters by Zone.

		BD	BH	FAR	SVF	FAI_1	FAI_2	FAI_3	FAI_4
Zone 1	Avg	0.19	12.01	1.2	0.93	0.07	0.07	0.07	0.06
	Min	0.001	3	0.001	0.47	0.001	0.001	0.001	0.001
	Max	0.76	105.27	10.73	1	0.55	0.52	0.52	0.41
Zone 2	Avg	0.28	11.48	1.65	0.93	0.09	0.08	0.08	0.07
	Min	0.001	3	0.001	0.6	0.001	0.001	0.001	0.001
	Max	0.69	67.13	9.86	1	0.38	0.31	0.31	0.29
Zone 3	Avg	0.21	15.72	1.72	0.9	0.11	0.11	0.11	0.09
	Min	0.001	3	0.001	0.47	0.001	0.001	0.001	0.001
	Max	0.71	105.27	10.73	1	0.55	0.52	0.52	0.41
Zone 4	Avg	0.18	10.48	0.97	0.94	0.06	0.06	0.06	0.05
	Min	0.001	3	0.001	0.57	0.001	0.001	0.001	0.001
	Max	0.76	93	9.85	1	0.42	0.41	0.41	0.37

Note: FAI_1, FAI_2, FAI_3, and FAI_4 indicate FAI values for spring, summer, autumn, and winter, respectively.

Table S4. Statistics on NDVI data by Zone.

	Zone 1			Zone 2			Zone 3			Zone 4		
	Avg	Min	Max	Avg	Min	Max	Avg	Min	Max	Avg	Min	Max
Spring	0.28	0.08	0.69	0.25	0.14	0.46	0.26	0.16	0.51	0.28	0.12	0.58
Summer	0.42	0.17	0.86	0.36	0.18	0.71	0.38	0.22	0.75	0.44	0.2	0.8
Autumn	0.36	0.11	0.79	0.32	0.15	0.66	0.33	0.17	0.69	0.37	0.05	0.73
Winter	0.18	0.01	0.67	0.16	0.08	0.45	0.16	0.05	0.36	0.18	0.07	0.46

Table S5-a. Parameters of Zone 1 GWR Model results.

	Zone 1							
	Spr_D	Spr_N	Sum_D	Sum_N	Aut_D	Aut_N	Win_D	Win_N
AICc	-2098	996.2	10053	46491.5	-7027.5	-2791.9	-10745	-218.6
R^2	0.964	0.977	0.966	0.866	0.972	0.979	0.956	0.986
Adj.R^2	0.954	0.97	0.956	0.826	0.963	0.972	0.943	0.982
Adj.α	0.000	0.000	0.000	0.000	0.000	0.000	0.000	0.000
Adj.t	3.909	3.909	3.909	3.895	3.909	3.909	3.911	3.911

Table S5-b. Parameters of Zone 2 GWR Model results.

	Zone 2							
	Spr_D	Spr_N	Sum_D	Sum_N	Aut_D	Aut_N	Win_D	Win_N
AICc	-191	1253	-71.1	1672	-858.5	403.6	-102.8	464.3
R^2	0.923	0.917	0.94	0.887	0.935	0.914	0.884	0.902
Adj.R^2	0.899	0.892	0.921	0.852	0.915	0.887	0.849	0.871
Adj.α	0.001	0.001	0.001	0.001	0.001	0.001	0.001	0.001
Adj.t	3.248	3.243	3.25	3.25	3.251	3.246	3.249	3.251

Table S5-c. Parameters of Zone 3 GWR Model results.

	Zone 3							
	Spr_D	Spr_N	Sum_D	Sum_N	Aut_D	Aut_N	Win_D	Win_N
AICc	-2603.4	988.1	668.5	17509	-4215.7	243.8	-4149.9	1187.5
R^2	0.951	0.969	0.96	0.843	0.958	0.963	0.913	0.959
Adj.R^2	0.936	0.959	0.947	0.794	0.945	0.951	0.885	0.945
Adj.α	0.000	0.000	0.000	0.000	0.000	0.000	0.000	0.000
Adj.t	3.608	3.608	3.608	3.587	3.609	3.604	3.61	3.61

Table S5-d. Parameters of Zone 4 GWR Model results.

	Zone 4							
	Spr_D	Spr_N	Sum_D	Sum_N	Aut_D	Aut_N	Win_D	Win_N
AICc	52192.7	51408	52715.6	52041.9	52291.8	51475	51272.4	50613.3
R^2	0.642	0.647	0.647	0.648	0.634	0.639	0.644	0.65
Adj.R^2	0.544	0.55	0.549	0.551	0.534	0.54	0.545	0.554
Adj.α	0.000	0.000	0.000	0.000	0.000	0.000	0.000	0.000
Adj.t	3.689	3.688	3.689	3.681	3.688	3.688	3.689	3.689

Notes: Spr_D, Spr_N, Sum_D, Sum_N, Aut_D, Aut_N, Win_D and Win_N denote spring day, spring night, summer day, summer night, autumn day, autumn night, winter day and winter night, respectively.

Table S6. OLS Model Accuracy.

	Zone 1		Zone 2		Zone 3		Zone 4	
	R^2	Adj.R^2	R^2	Adj.R^2	R^2	Adj.R^2	R^2	Adj.R^2
Spr_D	0.18	0.18	0.029	0.023	0.148	0.147	0.179	0.178
Spr_N	0.207	0.207	0.168	0.164	0.119	0.118	0.199	0.198
Sum_D	0.149	0.149	0.043	0.038	0.1	0.109	0.088	0.087
Sum_N	0.016	0.016	0.166	0.161	0.007	0.005	0.047	0.046
Aut_D	0.156	0.155	0.014	0.008	0.119	0.118	0.157	0.156
Aut_N	0.215	0.214	0.151	0.146	0.109	0.108	0.165	0.164
Win_D	0.078	0.078	0.051	0.046	0.009	0.008	0.085	0.084
Win_N	0.224	0.224	0.1	0.095	0.082	0.08	0.148	0.147
